# Evaluation of different diagnostic methods for the detection of *Mycobacterium avium* subsp. *paratuberculosis* in boot swabs and liquid manure samples

**DOI:** 10.1186/s12917-017-1173-6

**Published:** 2017-08-18

**Authors:** Nathalie Hahn, Klaus Failing, Tobias Eisenberg, Karen Schlez, Peter-Michael Zschöck, Karsten Donat, Esra Einax, Heike Köhler

**Affiliations:** 1 Animal Health Service, Thuringian Animal Diseases Fund, Jena, Thuringia Germany; 2 Unit for Biomathematics and Data Processing, Justus-Liebig-University, Gießen, Hesse Germany; 3 Department Veterinary Medicine, Hessian State Laboratory, Gießen, Hesse Germany; 4 Institute for Molecular Pathogenesis, Friedrich-Loeffler-Institut, Federal Research Institute for Animal Health, Jena, Thuringia Germany

**Keywords:** Cultural isolation, Diagnostic performance, DNA extraction, Environmental samples, qPCR

## Abstract

**Background:**

Environmental sampling based on boot swabs and/or liquid manure samples is an upcoming strategy for the identification of paratuberculosis (paraTB) positive herds, but only limited data are available regarding the diagnostic performance of molecular detection methods (qPCR) versus faecal culture (FC) for this purpose. In the present study, the test characteristics of two different qPCR protocols (A and B) and a standardized FC protocol, for the detection of *Mycobacterium avium* subsp. *paratuberculosis* in boot swabs and liquid manure samples were evaluated.

**Results:**

In 19 paraTB unsuspicious and 58 paraTB positive herds boot swabs and liquid manure were sampled simultaneously and analyzed in three different diagnostic laboratories. Using boot swabs and liquid manure, a substantial to excellent accordance was found between both qPCRs, for boot swabs also with culture, while for liquid manure the detection rate of culture was decreased after prolonged storage at −20 °C. The quantitative results of both qPCR methods correlated well for the same sample and also for boot swabs and liquid manure from the same herd. When cut-off threshold cycle (C_T_-)-values were applied as recommended by the manufacturers, herd level specificity (Sp) of qPCR B was below 100% for boot swabs and for both qPCRs for liquid manure. A decreased herd level sensitivity was encountered after adjustment of Sp to 100% and re-calculation of the cut-off C_T_-values.

**Conclusions:**

qPCR is equally suitable as bacterial culture for the detection of *Mycobacterium aviu*m subsp. *paratuberculosis* in boot swabs and liquid manure samples. Both matrices represent easily accessible composite environmental samples which can be tested with reliable results. The data encourage qPCR testing of composite environmental samples for paraTB herd diagnosis.

## Background

Environmental sampling constitutes an upcoming strategy for the identification of paratuberculosis (paraTB)-positive dairy herds or for the classification of the paraTB herd status because of reasonable expenses and the simple sampling technique [1–3]. Sampling six composite environmental faecal samples from manure concentration areas and manure storage areas is one option for herd classification within the Voluntary Bovine Johne’s Disease Control Program (VBJDCP) in the US [4]. Using this number of samples per holding, the sensitivity of the technique is not significantly influenced by the sampled locations, while reduction of the sample number without loss of sensitivity demands proper selection of the sampled areas, preferably alleyways and manure lagoons, where manure from numerous cows accumulates and is well mixed [5]. Recently, the boot swab (BS) sampling technique which is established for *Salmonella* testing in poultry flocks was adapted for environmental sampling of *Mycobacterium avium* subsp. *paratuberculosis* (MAP) in dairy herds, an approach overcoming the issue of sampling location and further reducing effort and cost [6]. The detection limit of this approach in terms of within-herd prevalence (WHP) is low [7], but depends on the laboratory methods used to substantiate MAP in the samples. Faecal culture (FC), still considered the most sensitive method for MAP detection is time-consuming and prone to microbial contamination [8]. Molecular techniques utilizing nucleic acid amplification are time saving alternatives. Meanwhile, several end-point and more recently Real-Time PCR (qPCR) protocols with excellent analytical sensitivity based on different targets have been developed for the identification of MAP [9–15].

However, diagnostic sensitivity of molecular methods relative to cultural isolation depends on the extraction protocol used to isolate MAP DNA from the samples. Several factors influence the efficiency of DNA extraction: homogeneity of MAP distribution in the samples, adequate disruption of the mycobacterial cell wall, and removal of DNA amplification inhibitors [14, 16]. Different silica membrane-based and magnetic bead-based extraction protocols for MAP from faeces have been proposed [17–20]. Significant differences in the purity and yield of the obtained DNA have been reported. However, similar extraction efficiency can be achieved with optimized silica membrane-based as well as magnetic bead-based procedures [21]. The detection rate can be improved by including a microfiltration step in the sample pre-treatment [22].

Conflicting data exist regarding test performance of direct qPCR relative to cultural isolation for the detection of MAP in individual and pooled faecal samples. While some authors report a higher diagnostic sensitivity of direct qPCR [23–26], others found similar [27, 28], or even lower [29] detection rates of qPCR in comparison to culture.

A good agreement of quantitative results between both diagnostic methods was found when pooled faecal and environmental samples were examined, reflected in a good to excellent correlation between threshold cycle (C_T_-) value and colony forming unit (CFU) counts [30].

Because comparative data for BS and liquid manure (LM) are lacking the aim of the present study was to determine the diagnostic performance of two different direct qPCR protocols (one silica membrane-based, one magnetic bead-based) and faecal culture for these matrices. We hypothesize

1. that direct qPCR protocols are equally suitable for the detection of MAP in BS and LM samples as cultural isolation, and
2. that BS and LM samples are equally suitable for the detection of MAP in the barn environment of MAP infected cattle.

## Methods

### Study population

Seventy-seven dairy herds from Thuringia, Hesse and Saxony, all federal states of Germany, with different within herd prevalence of paraTB were selected for this study. Nineteen herds were certified as ‘paraTB-unsuspicious’ that is comparable to the ‘herd classification no. 6’ of the uniform program standards of the VBJDCP [4]. The other 58 herds were classified as MAP positive based on recent results of FC of individual faecal samples obtained for whole herd testing. All herds were housed in free stalls.

### BS and LM sampling

The BS samples were collected as described by Eisenberg et al. [6] by a veterinarian of the animal health service from each federal state. Briefly, three BS were taken in parallel mainly in localizations with high cow traffic involving manure concentration areas which had been proven to be suitable for environmental sampling [31].

Additionally, samples from LM storage areas (tanks, lagoons, pits or pre-flooders) were obtained as described recently by Donat et al. [7].

Three laboratories working under quality management standards were involved in the study (LI – LIII). After sampling, BS and LM were immediately transported to either LI or LII. LM was divided into three aliquots of equal volume. All samples were frozen and stored at −20 °C for 1 to 2 months except LM for qPCR B. For technical reasons these latter samples were stored for 8 to 10 months at −20 °C. One BS and one aliquot of LM of each herd were shipped frozen to the two other laboratories.

FC of all samples was performed in the three laboratories. Furthermore, the samples were tested by two different qPCR methods, qPCR A (LII) and qPCR B (LIII).

### Boot swab handling

BS were placed in 125 mL plastic cups with screw cap and cut into small pieces with sterile scissors. In LI and LII, 100 mL of sterile physiologic salt solution (Merck, Darmstadt, Germany) were added and faecal material was rinsed off the boot swab pieces by automatically shaking at 200 rpm for 30 min. The eluate was transferred into a sterile 50 mL centrifuge tube (Sarstedt, Nümbrecht-Elsenroth, Germany). The supernatant was discarded after centrifugation for 15 min at 2000 x g.

In LIII, 3 g of cut BS were directly processed for bacterial culture and qPCR as described below.

### Bacterial culture

FC of processed BS as well as LM was done according to the official manual of diagnostic procedures published by the Friedrich-Loeffler-Institut, the German Federal Research Institute of Animal Health [32].

First, 3 g of faecal material or 5 g of cut BS (LIII) were mixed with 30 mL of a 0.75% hexadecylpyridinium chloride solution (Acros Organics, Fisher Scientific GmbH, Nidderau, Germany) for decontamination. After sedimentation of coarse material for 5 min, 20 mL of the supernatant were decanted, horizontally shaken for 30 min at 200 rpm and subsequently incubated for 48 h at room temperature in the dark. Then, the supernatant was discarded and 0.2 mL of the sediment was inoculated onto each of 3 tubes of commercial Herrold’s Egg Yolk Medium (HEYM) with mycobactin J, amphotericin B, nalidixic acid and vancomycin (ANV; Becton Dickinson, Heidelberg, Germany). After cultivation under aerobic conditions (37 °C ± 2 °C) for 7 days in a slanting position, the tubes were locked airtight and incubated vertically for another 11 weeks. Differentiation of suspected colonies was carried out by IS*900* PCR [33]. Samples were characterized as MAP-positive (MAP+), MAP-negative (MAP-) or non-assessable (n.a.) due to microbial contamination. The number of cultivable bacteria was recorded semi-quantitatively as a score; score 1: up to 10 colonies/tube, score 2: 11–50 colonies/tube, score 3: 51–100 colonies/tube, score 4: >100 colonies/tube. The average score of the three tubes per sample was calculated.

### DNA preparation and PCR

#### qPCR A, *utilizing nucleic acid purification with magnetic beads*

Nucleic acids were extracted from faeces using the MagMax™ Total NucleicAcid isolation kit (Life Technologies, Darmstadt, Germany) according to the instruction of the manufacturer, which uses zirconia beads for the mechanical disruption and magnetic beads for nucleic acid purification.

The samples were thawed, homogenized and 0.3 g were transferred into 1 mL phosphate buffered saline. After homogenization by vortexing for 3 min and centrifugation at 100 x g for 60 s, 175 μl of the supernatant were added to the zirconia bead tubes previously filled with 235 μl lysis/binding solution. The samples were homogenized three times for 30 s at 6800 rpm (Precellys® 24 homogenizer, Bertin Technologies, Montigny-le-Bretonneux, France) and centrifuged at 16,000 x g for 3 min.

To clarify the lysate, 300 μl of the sample were transferred into a new 1.5 mL tube and centrifuged for a second time at 16,000 x g for 6 min.

The MagMax™ Express 96 instrument (Life Technologies) was applied for nucleic acid purification. After four washing steps the purified DNA was directly used for the qPCR.

For the detection of MAP DNA, the VetMAX™ MAP Real-Time PCR screening kit (Life Technologies), performed on a 7500 Fast qPCR cycler (Life Technologies), was used according to the instruction of the manufacturer.

A sample was detected to be positive at C_T_-values ≤37.0, inconclusive at C_T_-values >37.0 and <40.0. Samples with atypical amplification curves or with undetermined C_T_-values for target and internal control (PCR inhibition) were considered n.a.

#### qPCR B*, utilizing sample enrichment and silica membrane-based nucleic acid purification*

5.0 g of cut BS or 3.0 g of LM, respectively, were suspended in 20 mL sterile distilled water and left for 10 to 20 min at room temperature for sedimentation of coarse material. To concentrate bacteria and remove PCR inhibiting substances, 10 mL of the supernatant were centrifiltrated using Adiafilter (ADIFIL 100, Adiagene, Saint Brieux, France) and the pellet was re-dissolved in 500 μL sterile distilled water. Bacterial cells were mechanically disrupted by bead-beating with 300 mg glas beads (Peqlab, Erlangen, Germany) for 10 min at 30 Hz using the TissueLyser (Qiagen, Hilden, Germany). After an initial centrifugation step (5 min, 15,000 x g) the nucleic acid extraction was carried out using the QIAamp® DNA Mini kit (Qiagen) according to the instruction of the manufacturer. The qPCR was performed with the Adiavet™ Paratb Real time kit (Adiagene) using the 7500 Real Time PCR system (Applied Biosystems, Foster City, CA, USA) as recommended. Samples were tested in duplicate and the mean C_T_-value of both replicates was calculated. A sample was detected to be positive at mean C_T_-values ≤38.0, inconclusive at mean C_T_-values >38.0 and ≤40.0 and n.a. when one replicate yielded a measurable and the other an undetermined C_T_-value for the target.

### Statistical data analysis

Data recording and descriptive statistics were performed using a Microsoft Excel^®^ spreadsheet (Microsoft Office 2007, Microsoft Corporation, Redmond, USA). Further statistical examinations were done by means of the statistical programme packages BMDP [34] and StatXact 9.0 [35]. The figures were created with the statistical software package PASW Statistics 17 (SPSS, Quarry Bay, Hongkong).

For each matrix, BS and LM samples, the Spearman’s rank correlation coefficient (programme BMDP3D) was calculated in order to analyse the relationship between the semi-quantitative colony growth score of FC and the C_T_-values of qPCR.

Kappa coefficients were calculated with the programme BMDP4F to describe the degree of agreement between the dichotomized outcomes (negative or positive) of the FC and the qPCR test systems. The following levels of agreement were considered for the interpretation of the kappa coefficient (ĸ) [36]: < 0.20: slight; 0.21–0.40: low; 0.41–0.60: moderate; 0.61–0.80: substantial; 0.81–1.00: excellent. Furthermore, both calculations were performed for the description of the accordance between qPCR A and B for each matrix, and for the accordance between BS and LM using either PCR A or B. Herd level Se and Sp for both qPCR test systems applied either on BS or LM samples were estimated by a frequency-based approach classifying herds according to their certification status (MAP+, MAP-) using the cut-off C_T_-values as recommended by the manufacturers for faecal samples. The corresponding exact 95% confidence intervals of the binomial distribution were calculated according to Clopper and Pearson [37]. Cut-offs for both qPCR tests were estimated by TG-ROC-analysis [38, 39]. Herd level Se and the cut-offs of the qPCRs were re-calculated after adjusting the values for herd level Sp to 100%. These calculations were performed using R software [40], version 2.15.2 (2012–10-26), with package DiagnosisMed [41]. In general, the statistical significance level was set at α = 0.05.

## Results

A total of 77 pairs of BS and LM samples were tested in parallel by FC in the three laboratories. Additionally, all samples were analyzed by one of two different direct qPCR methods. The proportion of culture positive samples was comparable between the three laboratories and between BS and LM, except for LM in LIII, where a markedly lower number of samples were tested positive (see Table 1). LM tended to a higher number of n.a. samples (due to fungal overgrowth) than BS, although the proportion was low in both matrices (BS 0–1.3%, LM 2.6%). Compared to cultural isolation, with qPCR the number of positive BS and LM was slightly lower and the proportion of n.a. samples was higher, especially when LM were analyzed (Table 1). The reasons for n.a. results differed between the two qPCR protocols. When qPCR A was applied they were mainly due to inhibition. With qPCR B the samples contained only very low amounts of target DNA leading to inconsistent results of the two replicates. A substantial to excellent accordance was observed between the results (positive or negative) of FC and qPCR for BS within the two laboratories and in LII also for LM (Table 2). In LIII, due to the reduced number of culture-positive LM samples (Table 1), only a moderate accordance between the results of both methods could be achieved (Table 2). Semi-quantitative FC results and qPCR C_T_-values were significantly negative correlated with concordant Spearman rank correlation coefficients (r_s_) for BS (qPCR A − 0.878; qPCR B − 0.889) and LM (qPCR A − 0.886; qPCR B − 0.730) (Table 2).

**Table 1:** Results of the analysis of boot swabs and liquid manure samples (n/ %) from 77 herds by faecal culture in three labs (I to III) and by two different qPCR methods (qPCR A and qPCR B) for *Mycobacterium avium* ssp. *paratuberculosis* (MAP)

Methode of MAP detection	Lab^a^	Boot swab			Liquid manure		
		pos^b^	neg^c^	n.a.^d^	pos	neg	n.a.
Faecal culture	I	40/ 51.9	37 /48.0	0 /0.0	39 /50.6	36 /46.8	2 /2.6
Faecal culture	II	41/ 53.2	36 /46.8	0 /0.0	38 /49.4	37 /48.0	2 /2.6
Faecal culture	III	41 /53.2	35 /45.5	1 /1.3	21 /27.3	54 /70.1	2 /2.6
qPCR A	II	35 /45.5	39 /50.6	3 /3.9	33 /42.9	35 /45.5	9 /11.7
qPCR B	III	37 /48.0	36 /46.8	4 /5.2	38 /49.4	32 /41.6	7 /9.1

^a^Laboratory

^b^MAP-positive

^c^MAP-negative

^d^Non-assessable faecal culture due to contamination or non-assessable qPCR results

**Table 2:** Accordance between the results of faecal culture and the two qPCRs for boot swabs and liquid manure samples

Matrix	qPCR	r_s_^a,b^	ĸ ± SE_app_^c^
Boot swab	A	- 0.878	0.757 ± 0.076
	B	- 0.889	0.808 ± 0.069
Liquid manure	A	- 0.866	0.794 ± 0.074
	B	- 0.730	0.514 ± 0.102

^a^Spearman rank correlation coefficient

^b^p-value for all Spearman rank correlation coefficients: *P* < 0.0001

^c^Cohen’s kappa coefficient ± approximate standard error

**Fig. 1:**
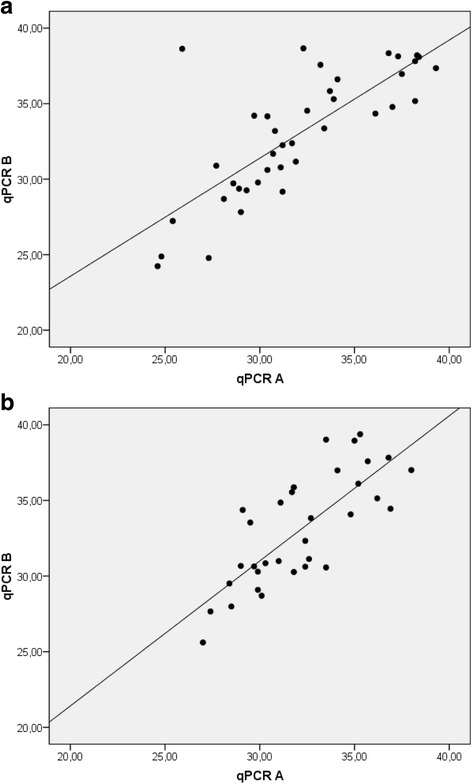
Correlation diagram of the C_T_-values of qPCR A and B for test positive samples. **a** using boot swabs and **b** using liquid manure samples. Included are only samples with positive qPCR results in both tests based on the cut-off values as recommended by the manufacturers’

The test performance of the two qPCRs in terms of positive and negative outcome was comparable as reflected in Cohen’s kappa values which demonstrate a substantial accordance for BS (κ: 0.629 ± 0.092) and LM (κ: 0.688 ± 0.090). The quantitative results of qPCR A and B correlated well, with r_s_ of 0.886 (BS) and 0.885 (LM) (Fig. 1a and b).

Using qPCR A test results (positive or negative) for both specimens, BS and LM, from one herd corresponded clearly (κ: 0.784 ± 0.077), while the accordance between the results of qPCR B for both matrices was less pronounced (κ: 0.543 ± 0,104) (Table 3). However, the quantitative qPCR results (C_T_-values) of BS and LM from the same herd correlated markedly for qPCR A (r_s_: 0.845) and qPCR B (r_s_: 0.779).

**Table 3:** Accordance between matrices, boot swab and liquid manure, using qPCR A or qPCR B

qPCR	r_s_^a,b^	ĸ ± SE_app_^c^
A	0.845	0.784 ± 0.077
B	0.779	0.543 ± 0.104

^a^Spearman rank correlation coefficient

^b^p-value for all Spearman rank correlation coefficients: *P* < 0.0001

^c^Cohen’s kappa coefficient ± approximate standard error

Herd level Se of qPCR B was slightly higher than that of qPCR A for both matrices, when cut-off C_T_-values were applied as recommended by the manufacturers. However, under these conditions, the herd level Sp of qPCR B for BS and of qPCR A and B for LM was below 100% (Table 4), leading to false positive test results. Re-calculation after adjustment of Sp to approximately 100% resulted in decreased cut-off C_T_-values and, consequentially, in a reduced Se for these test-specimen combinations (Table 5).

**Table 4:** Test characteristics of qPCR A and B for analysis of boot swabs and liquid manure samples using the cut-off C_T_-values as recommended by the manufacturers’ for faecal samples

Matrix	qPCR	Cut-off C_T_^a^	Test + /Status +^b^	Se [%] (95% CI)	Test - /Status -^c^	Sp [%] (95% CI)
Boot swab	A	37.00	35/57	61.40 (47.57–74.00)	17/17	100.00 (80.49–100.00)
	B	38.00	36/55	65.45 (51.42–77.76)	16/17	94.44 (72.71–99.86)
Liquid manure	A	37.00	32/51	62.75 (48.08–75.87)	16/17	94.12 (71.31–99.85)
	B	38.00	36/53	67.92 (53.68–80.08)	15/17	88.24 (63.56–98.54)

^a^C_T_-values ≤37.00 (qPCR A) and ≤38.00 (qPCR B) were considered positive and C_T_-values >37.00 (qPCR A) and >38.00 (qPCR B) were considered negative.

^b^Number of test positive samples per total number of assessable samples from MAP-positive herds (n/n).

^c^Number of test negative samples per total number of assessable samples from MAP-negative herds (n/n)

**Table 5:** Results of the TG-ROC analysis: Cut-off C_T_-values and Se of qPCR A and B when Sp was adjusted to 100.0%

Matrix	qPCR	Cut-off C_T_	Se [%] (95% CI)	Sp [%]^a^ (95% CI)
Boot swab	A	39.30	73.68 (60.34–84.46)	100.00 (80.49–100.00)
	B	36.12	50.91 (37.07–64.65)	100.00 (81.47–100.00)
Liquid manure	A	35.10	50.98 (36.60–65.25)	100.00 (80.49–100.00)
	B	35.99	50.94 (36.84–64.94)	100.00 (80.94–100.00)

^a^Sp was fixed at 100.0%, the corresponding Se and cut-offs were calculated

## Discussion

The present data support the concept that qPCR is equally suitable for the detection of MAP in composite environmental samples as bacterial culture on solid media. Recently, it has been shown that use of qPCR can yield rapid, quantitative estimates of MAP load in pooled faecal and composite environmental samples that were collected according to the recommendations of the USDA National Animal Health Monitoring System (NAHMS) [30]. However, in this study, qPCR was applied to BS for the first time, and test performance on samples from paraTB-unsuspicious herds has not been studied so far.

The herd level Sp of both qPCR methods was below 100% when cut-off C_T_ values were applied as to the recommendations of the manufacturers. Under these conditions, positive PCR results in supposedly paraTB-unsuspicious herds could have different reasons: (1) a very low WHP of MAP-infected animals which was not detected before or, (2) cross-contamination of samples during processing from sample collection in the herd to DNA extraction and PCR set up in the laboratory. It cannot be excluded that single very low shedders have been overlooked in herds with a history of complete negative results of bacterial culture on the individual animal level. Moreover, cross contamination during PCR workflow which has only rarely been addressed in the literature [42–44], has to be considered, particularly when samples are collected in the typical environment of dairy cattle herds and when sample preparation protocols comprise several manual handling steps. Semi-automated sample preparation seems to be less prone to cross contamination, because qPCR A, for which such a DNA extraction protocol was applied, had a higher Sp than qPCR B. However, the results have to be interpreted with caution because the number of paraTB-unsuspicious herds in this study (*n* = 19) was rather low.

Both qPCR methods are equally suitable for the examination of environmental samples as bacterial culture as reflected by the substantial to excellent accordance between both methods for BS (both qPCRs) and LM (qPCR A). Storage of LM samples at −20 °C for several months resulted in a decreased detection rate of bacterial culture (Table 1), leading to a lower Cohen’s κ value (0.514 ± 0.102) and reduced correlation of the quantitative results (r_s_: - 0.730, Table 2). This confirms data from Aly et al. [30] and points to the fact that MAP detection by qPCR is less affected by prolonged frozen sample storage than detection of viable bacteria by culture. However, the proportion of n.a. test results of both qPCR methods was higher than for faecal culture. Reasons were, on the one hand, samples with divergent results because two replicates per sample were analyzed (qPCR B). We assume that even a very low content of target DNA which is irregularly distributed in the DNA extract is detected because of the high analytical Se of the method. Re-sampling and testing of the respective herds may result in a reliable herd classification. On the other hand, n.a. qPCR results were due to complete or incomplete inhibition of amplification (qPCR A), reflected by no or non-typical amplification curves. It has been shown that inhibition is relieved by dilution of the DNA extract [45], which is also recommended by the qPCR manufacturer but was not done in the present study. Generally, LM were more affected by n.a. results than BS. It seems that in LM i) dilution of MAP positive faeces is more probable and ii) the mixture of faecal material with urine and other effluents increases the risk of qPCR inhibition.

The excellent (qPCR A) or substantial (qPCR B) accordance between the results of BS and LM indicates that both matrices can be reliably examined by qPCR. Combination of BS and LM leads to an even higher detection rate than testing only one matrix [7]. Cohen’s κ value of qPCR B (0.543, Table 3) was lower than that of qPCR A (0.784). This is due to the fact that qPCR B detected a higher proportion of samples with low bacterial load and therefore, frequently only one of the two matrices was positive, leading to lower accordance and a lower κ value. In contrast, the excellent accordance between BS and LM when tested with qPCR A is due to a lower sensitivity of the method causing a higher proportion of negative qPCR results in samples with low bacterial load. This applies predominantly to samples from herds with low WHP [7]. A good correlation between the C_T-_values of BS and LM was noted for both qPCRs. This shows that the MAP load in LM is related to the MAP load in the herd environment which in turn is dependent on WHP [7].

Despite a slightly higher Se of qPCR B both PCRs showed comparable results. Cohen’s κ values point to a moderate accordance. qPCR B encompasses a DNA extraction protocol that includes a microfiltration step in combination with silica membrane-based mini-columns which has been shown to be one of the most sensitive protocols [22].

Using the cut-off C_T-_values as recommended by the manufacturers, Sp and Se of both qPCR’s for both matrices are comparable to each other (Table 4). As the definition of the herd status MAP+ based on herd history including also herds without actual shedders at the day of environmental sampling, the estimates for sensitivity are rather conservative in our study. Similar values have been achieved when a set of composite environmental samples was examined by bacterial culture [3] as recommended by the USDA for herd classification [4].

Only a low number of paraTB-unsuspicious herds was included in the present study (*n* = 19). Therefore, only a few false positive test results account for a considerable drop of Sp of the methods. As already discussed above, several reasons for false positive test results have to be considered. Altogether, a larger proportion of paraTB-unsuspicious herds is necessary to verify the Sp results of both qPCRs for BS and LM. Adjustment of Sp to about 100% and re-calculation of the cut-off C_T-_values resulted in diminished Se, because samples with high C_T-_values were classified negative, except for qPCR A when boot swabs were examined. For this test configuration, re-calculation resulted in a higher cut-off C_T-_value, and Se was not altered, because no false positive samples existed. As discussed for Sp, a few false positive test results account for marked shifts of the re-calculated cut-off C_T-_values and, therefore, these cut-offs cannot be recommended for practical use.

## Conclusions

qPCR is equally suitable as bacterial culture for the detection of MAP in composite environmental samples. Boot swab and LM samples represent easily accessible composite environmental samples which can be tested with reliable results. Examination of larger numbers of herds is necessary to fix valid cut-off C_T-_values for qPCR methods which ensure a high herd level Sp in combination with a high diagnostic Se.

## Abbreviations

ANV: Amphotericin B, nalidixic acid, vancomycin
BS: Boot swab(s)
CFU: Colony forming units
C_T_: Threshold cycle
FC: Faecal culture
HEYM: Herrold’s Egg Yolk medium with mycobactin and ANV
LM: Liquid manure
MAP: *Mycobacterium avium* subsp. *paratuberculosis*
n.a.: Non-assessable
paraTB: Paratuberculosis
qPCR: Quantitative polymerase chain reaction
r_s_: Spearman rank correlation coefficient
Se: Sensitivity
Sp: Specificity
WHP: Within-herd prevalence
κ: kappa
